# A Signal-Processing-Based Approach to Time-Varying Graph Analysis for Dynamic Brain Network Identification

**DOI:** 10.1155/2012/451516

**Published:** 2012-08-07

**Authors:** Ali Yener Mutlu, Edward Bernat, Selin Aviyente

**Affiliations:** ^1^Department of Electrical and Computer Engineering, Michigan State University, East Lansing, MI 48824, USA; ^2^Department of Psychology, Florida State University, Tallahassee, FL 32306, USA

## Abstract

In recent years, there has been a growing need to analyze the functional connectivity of the human brain. Previous studies have focused on extracting static or time-independent functional networks to describe the long-term behavior of brain activity. However, a static network is generally not sufficient to represent the long term communication patterns of the brain and is considered as an unreliable snapshot of functional connectivity. In this paper, we propose a dynamic network summarization approach to describe the time-varying evolution of connectivity patterns in functional brain activity. The proposed approach is based on first identifying key event intervals by quantifying the change in the connectivity patterns across time and then summarizing the activity in each event interval by extracting the most informative network using principal component decomposition. The proposed method is evaluated for characterizing time-varying network dynamics from event-related potential (ERP) data indexing the error-related negativity (ERN) component related to cognitive control. The statistically significant connectivity patterns for each interval are presented to illustrate the dynamic nature of functional connectivity.

## 1. Introduction

 The human brain is known to be one of the most complex systems and understanding its connectivity patterns for normal and disrupted brain behavior remains as a challenge. Over the last decade, there has been a growing interest in studying brain connectivity. In literature, three kinds of brain connectivity have been addressed to define interactions between different regions of the human brain: anatomical connectivity, functional connectivity, and effective connectivity [1, 2]. Anatomical connectivity is defined as the set of connections at the physical or structural layer which links neuronal units at a given time and can be analyzed using techniques such as diffusion tensor imaging [3, 4]. Functional connectivity is defined as the statistical dependencies among remote neurophysiological events, which indicate the integration of functionally segregated brain regions. Finally, effective connectivity refers to causal relations between neural systems where causality is understood in at least two distinct ways: temporal precedence and physical influence [5–7]. In this paper, we limit our focus on discovering functional connectivity where reciprocal interactions are investigated.

Functional connectivity can be inferred from different neuroimaging data such as the functional magnetic resonance imaging (fMRI), electroencephalography (EEG), and magnetoencephalography (MEG) [8]. fMRI provides a high spatial resolution whereas EEG, and MEG have more limited spatial resolution. However, EEG and MEG offer higher temporal resolution required for quantifying the time-varying relationships between neuronal oscillations compared to fMRI which makes these recording techniques more appealing for quantifying the functional brain connectivity. Various measures, such as spectral coherence and phase synchrony, have been proposed for quantifying the functional relationships among different brain regions [9]. However, these measures are limited to quantifying pairwise relationships and cannot provide an understanding of the collective behavior of different brain regions. Attempts to characterize the topologies of these large networks led to the emergence of a new, multidisciplinary approach to the study of complex systems based on graph theory, which has been used to analyze models of neural networks, anatomical connectivity, and functional connectivity based upon fMRI, EEG and MEG. Network characterization of functional connectivity data is motivated by the development of neurobiologically meaningful and easily computable measures, such as graph theory-based clustering coefficient and characteristic path length, that reliably quantify brain networks [1, 10–13]. These measures also offer a simple way to compare functional network topologies between subject populations and have been shown to reveal presumed connectivity abnormalities in neurological and psychiatric disorders [14, 15].

A network is a mathematical representation of a real-world complex system with relational information and can be represented by a graph consisting of a set of vertices (or nodes) and a set of edges (or connections) between pairs of nodes. The presence of a connection between two vertices means that there is some kind of relationship or interaction between the nodes. In order to emphasize the strength of the connectivity between nodes, one can assign weights to each of the edges and the corresponding graph is called a weighted graph. In the study of functional brain networks, nodes represent the different brain regions and the edges correspond to the functional connectivity between these nodes which are usually quantified by the magnitudes of temporal correlations in activity. Depending on the measure, functional connectivity may reflect linear interactions such as correlation or nonlinear interactions such as phase synchrony [9]. Graph theory provides a way to capture the topology of this network and to quantify the multivariate relationships among neuronal activations across brain regions as well as to suggest models for functional brain networks which may allow us to better understand the relation between network structure and the processes taking place on these networks. One such model is the “small-world” network introduced by Watts and Strogatz [16], that demonstrates both clustered “cliquish” interconnectivity within groups of nodes (like regular lattices) and a short path length between any two nodes (like random graphs). This is an attractive configuration for the functional architecture of the brain, because small-world networks are known to optimize information transfer, increase the rate of learning, and support both segregated and distributed information processing. Recently, there have been multiple functional network studies using graph theory based on fMRI [17], EEG [14, 18], and MEG data [12, 19] which have shown small-world patterns in functional networks of healthy subjects. Several studies have also shown how brain pathology, such as schizophrenia and Alzheimer's diseases, may interfere with the normal small-world architecture [10–12, 14, 20].

Currently, topological features of functional brain networks such as clustering coefficient, path length, small world parameter [21], modularity, global, and local efficiency are defined over long periods of time, thus focusing on static networks and neglecting possible time-varying properties of the topologies [22–24]. This consideration might be reasonable for anatomical connectivity; however, a single graph is not sufficient to represent the communication patterns of the brain and can be considered as an unreliable snapshot of functional connectivity. Evidence suggests that the emergence of a unified neural process is mediated by the continuous formation and destruction of functional links over multiple time scales [21].

In recent years, there has been an interest in characterizing the dynamic evolution of functional brain networks. Most of the existing approaches to dynamic network analysis are either graph theory based such as direct extensions of component finding [25–27] and community detection [28] from the static to the dynamic case or are feature based where features extracted from each graph in the time series are used to form time-varying graph metrics [29, 30]. This extension to dynamic networks reveals that the processing of a stimulus involves optimized functional integration of distant brain regions by dynamic reconfiguration of links. More recently, the dynamic nature of the modular structure in the functional brain networks has been investigated by finding modules for each time window and comparing the modularity of the partitions across time [31]. However, this approach does not evaluate the dynamic evolution of the clusters across time and is basically an extension of static graph analysis for multiple static graphs. Mucha et al. [28] proposed a new time-varying clustering algorithm which addresses this issue by defining a new modularity function across time. All of these module finding algorithms result in multiple clustering structures across time and there is a need to reduce this multitude of data into a few representative networks or to quantify the evolution of the network in time using reliable metrics. Therefore, these approaches do not track the change in connectivity or clustering patterns and cannot offer meaningful summarizations of time-varying network topology.

Recently, researchers in signal processing have addressed problems in dynamic network analysis such as detection of anomalies or distinct subgraphs in large, noisy background [32] and tracking dynamic networks [33]. Simple approaches such as sliding window or exponentially weighted moving averaging have been proposed for inferring long-term information or trends [34, 35]. However, these methods have some disadvantages such as preserving historical affinities indefinitely, which makes the network topology denser as time evolves [34]. In this paper, we will contribute to this line of work by finding the event intervals in functional brain connectivity patterns, revealing the most relevant and informative information for each interval and summarizing brain network activity with a few number of representative networks, similar to data reduction in signal processing where the ideal summary should conserve the minimum redundancy in representing the dynamics of the particular interval. Recently, similar data reduction problems in psychophysiological studies involving evoked brain potential activity across time, frequency, and space have been addressed [36, 37]. However, the work in this area focuses on reducing activation patterns across time, frequency, and space using a Bayesian classification approach [37]. Unlike this paper which considers the activation of each electrode individually in time- and frequency, our paper considers functional connectivity or multivariate relationships between electrode pairs and tries to reduce this relational information using a segmentation approach along time.

In this paper, we first construct time-varying graphs, which are needed to describe the brain activity across time, by quantifying the time-varying phase synchrony between different electrodes of the EEG data [38]. Then, a framework for summarizing or reducing the information in dynamic brain networks into a few representative networks will be proposed by computing the distances between subsequent graphs, detecting changes in distances to determine the event boundaries, and, finally, forming a key network for each interval such that this key network summarizes the particular interval with minimal redundancy.

## 2. Background

 Phase synchrony is defined as the temporal adjustment of the rhythms of two oscillators while the amplitudes can remain uncorrelated. The first step in quantifying the phase synchrony between two signals is to estimate the instantaneous phase of the individual signals, Φ_*i*_(*t*, *ω*), around the frequency of interest, *ω*. Once the phase difference, Φ_*i*,*j*_(*t*, *ω*) = |Φ_*i*_(*t*, *ω*) − Φ_*j*_(*t*, *ω*)|, between the two signals, *x*_*i*_ and *x*_*j*_, is estimated, phase synchronization can be quantified by means of the phase-locking value (PLV) which ranges in [0,1]:

(1) $$\begin{array}{c} {\text{PLV}}_{i,j}\left(t,\omega \right)=\frac{1}{K}\left|\sum_{k=1}^{N}\exp\left(j\Phi_{i,j}^{k}\left(t,\omega \right)\right)\right|, \end{array}$$

where *K* is the number of trials and Φ_*i*,*j*_^*k*^(*t*, *ω*) is the time-varying phase difference estimate for the *k*th trial. If the phase difference varies little across the trials, PLV is close to 1 which indicates high phase synchrony pair signals.

Two major approaches to extracting the instantaneous phase are the Hilbert transform and the complex wavelet transform. The Hilbert transform-based method obtains an analytic form of the signal and estimates the instantaneous phase from this analytic form [39]. However, one has to ensure that the signal is composed of a narrowband of frequencies and this requires the bandpass filtering of the signal around a frequency of interest which is followed by the application of the Hilbert transform to obtain the instantaneous phase. The second approach to phase synchrony computes a time-varying complex energy spectrum using the continuous wavelet transform (CWT) with a complex Morlet wavelet [40]. The main drawback of this measure is the nonuniform time-frequency tiling where the frequency resolution is high at low frequencies and low at high frequencies. Although this property is desirable in detecting high frequency transients in a given signal, it inherently imposes a non-uniform time-frequency resolution which results in biased energy and phase estimates. In this paper, we propose to use a new time-varying phase estimation method based on the Reduced Interference Rihaczek (RID-Rihaczek) distribution belonging to Cohen's class [38]. This distribution offers phase estimates with uniformly high time-frequency resolution which can be used for defining time- and frequency-dependent phase synchrony. Compared to the existing measures, in our previous work we have shown through both simulation and analysis that RID-Rihaczek-based phase and phase synchrony estimators are more robust to noise, have uniformly better time-frequency resolution with less bias in extracting time- and frequency-dependent phase, and perform superior at detecting actual synchrony within a group of oscillators [38].

It is important to note some limitations of PLV that have been investigated in recent work, specifically in the context of intertrial phase synchrony [41]. Specifically, the PLV cannot discriminate between additive versus phase-resetting activation in ERPs from trial to trial, and thus is not a reliable measure for studying event-related brain dynamics (ERBD [42]). Some recent approaches based on t-statistics type measures from complex time-frequency distribution coefficients offer some methods to decompose constituent contributions of amplitude and phase resetting to the PLV for intertrial measures [43]. However, it is not clear what role these components have in PLV when measuring functional connectivity.

### 2.1. RID-Rihaczek Distribution

 Rihaczek distribution is a complex time-frequency distribution that provides both a time-varying energy spectrum as well as a phase spectrum with good time-frequency localization for phase modulated signals [44] and is defined as

(2) $$\begin{array}{c} C_{i}\left(t,\omega \right)=\frac{1}{\sqrt{2\pi }}x_{i}\left(t\right)X_{i}^{*}\left(\omega \right)e^{-j\omega t}, \end{array}$$

where *x*_*i*_(*t*) is the signal and *X*_*i*_(*ω*) is its Fourier transform. The time- and frequency dependent phase estimate based on this distribution can be found as

(3) $$\begin{array}{c} \Phi_{i}\left(t,\omega \right)=\arg\;\left[\frac{C_{i}\left(t,\omega \right)}{\left|C_{i}\left(t,\omega \right)\right|}\right]=\phi_{i}\left(t\right)-\theta_{i}\left(\omega \right)-\omega t, \end{array}$$

where *ϕ*_*i*_(*t*) and *θ*_*i*_(*ω*) refer to the phase in the time and the frequency domains, respectively. Once the phase estimate in the time-frequency domain is obtained, the phase difference between two signals, *x*_*i*_(*t*) and *x*_*j*_(*t*), can be computed as

(4) Φi,j(t,ω)=arg [Ci(t,ω)|Ci(t,ω)|Cj∗(t,ω)|Ci(t,ω)|]=(ϕi(t)−ϕj(t))−(θi(ω)−θj(ω)).

For multicomponent signals, cross-terms occur at the same time- and frequency locations as the original signals and will lead to biased energy and phase estimates. In order to eliminate these cross-terms, we proposed a reduced interference version of the Rihaczek-distribution, which is referred to as RID-Rihaczek, by applying a Choi-Wiliams (CW) kernel function to filter the cross-terms in the ambiguity domain [45, 46]:

(5) Ci(t,ω)=∫∫exp⁡(−(θτ)2σ)︸CW  kernel exp⁡(jθτ2)︸Rihaczek  kernelA(θ,τ)e−j(θt+τω) dτdθ,

where exp⁡(*j*(*θτ*/2)) is the kernel function for the Rihaczek distribution and *A*(*θ*, *τ*) = ∫*x*_*i*_(*u* + *τ*/2)*x*_i_*(*u* − *τ*/2)*e* ^*jθu*^ *du* is the ambiguity function of the signal, *x*_*i*_(*t*).

## 3. Dynamic Network Summarization

 Let *G* = {*G*_*t*_}_*t*=1,2,…,*T*_ be a time sequence of weighted and undirected graphs where *G*_*t*_ is an *N* × *N* weighted and undirected graph at time *t*, *T* is the total number of time points, and *N* is the number of nodes within the network. The connectivity strength or the edge between nodes *i* and *j* at time *t* is represented by *G*_*t*_(*i*, *j*) and is in the range of [0,1].

We propose a dynamic graph summarization framework consisting of constructing time-varying graphs from pairwise phase synchrony measure, identifying event windows, revealing the most important and informative connectivity patterns to summarize each event window with a key graph and to describe the dynamic evolution of the network over time.

### 3.1. Forming Time-Varying Graphs via Phase Synchronization

In order to describe the evolution of time-varying connectivity patterns in the brain network, we first need to obtain the time-varying graphs. We quantify the bivariate relationship between nodes within the network and construct the time-varying graphs by considering the average synchrony within a frequency band at a certain time as

(6) $$\begin{array}{c} G_{t}\left(i,j\right)=\frac{1}{W}\sum_{\omega =\omega_{a}}^{\omega_{b}}{\text{PLV}}_{i,j}\left(t,\omega \right), \end{array}$$

where *G*_*t*_(*i*, *j*) represents the connectivity strength between the nodes *i* and *j* within the frequency band of interest, [*ω*_*a*_, *ω*_*b*_], and *W* is the number of frequency bins in that band. In this paper, our focus is to evaluate the dynamics of the networks over time and the proposed framework is designed accordingly. However, one can extend this framework to consider each time and frequency bin separately to evaluate the network changes over both time and frequency.

### 3.2. Event Interval Detection

Once the time-varying graphs are obtained, we need to identify meaningful time intervals which may account for the underlying neurophysiological events such as error-related negativity or Pe event-related potential elicited in the process of decision making. For this purpose, we propose to quantify the change in node *i*'s connectivity with other nodes from time point *t* to *t* + 1 as

(7) dt, t+1(i)=||gi(t+1)−gi(t)||∞=max⁡k {|gik(t+1)−gik(t)|}, k=1,2,…,N,

where *g*_*i*_^*k*^(*t*) is the *k*th element of *i*th row of *G*_*t*_ and *d*_*t*, *t*+1_(*i*) is in the range of [0,1]. *l*_*∞*_ norm highlights the maximum change in a node's connectivity from time *t* to *t* + 1 instead of the average change in the node's connectivity and, thus, is better at filtering out connections that are insignificant for that particular node. The average distance, *D*_*t*, *t*+1_, between the graphs *G*_*t*_ and *G*_*t*+1_ is then defined as

(8) $$\begin{array}{c} D_{t,\;t+1}=\frac{1}{N}\sum_{i=1}^{N}d_{t,\;t+1}\left(i\right). \end{array}$$

In order to detect the abrupt changes in the distance measure *D*_*t*, *t*+1_ at any time, we propose to employ a standard change detection algorithm based on adaptive thresholding:

(9) $$\begin{array}{c} I\left(t,t+1\right)=\{\begin{array}{cc} 1, & \text{if}\;\;\left|D_{t,\;t+1}-\mu_{t}\right|\geq 2\sigma_{t}\\ 0, & \text{if}\;\;\left|D_{t,\;t+1}-\mu_{t}\right|<2\sigma_{t}, \end{array} \end{array}$$

where an event boundary is detected, *I*(*t*, *t* + 1) = 1, depending on the deviation of *D*_*t*,*t*+1_ from the moving average, *μ*_*t*_ = (1/*δ*)∑_*k*=1_^*δ*^ *D*_*t*−*k*, *t*−*k*+1_. Adaptive thresholding value, 2*σ*_*t*_, is based on the standard deviation, $\sigma_{t}=\sqrt{\left(1/\delta \right)\sum_{k=1}^{\delta }{\left(D_{t-k,\;t-k+1}-\mu_{t}\right)}^{2}}$, and the length of the moving average window, *δ*, can be chosen based on the sampling frequency and total number of time samples, *T*.

### 3.3. Key Graph Estimation Using Principal Component Analysis

 After determining the event intervals, we need to form key graphs which best summarize the particular intervals. For this purpose, we need to distinguish between transient (high variance) and stationary (low variance) interactions within a given time interval and obtain a key graph which captures the transient or dynamic interactions. The ideal key graph should describe dynamic behavior of the particular interval with minimal redundancy. This is analogous to finding signal components that have low and high variance in a given data set and this separation in terms of variance is usually addressed through principal component analysis (PCA). Hence, we propose to employ PCA in order to extract key graphs and summarize the dynamics of the event intervals with minimal redundancy.

Let *G*_1_, *G*_2_,…, *G*_*M*_ be the set of *M* graphs that compose an event interval that we try to summarize. Since the graphs are undirected and symmetric, we create vectors, **z**_1_,…, **z**_M_, to equivalently represent the graphs where **z**_i_ is obtained by stacking the columns of the upper triangular portion of *G*_*i*_ and has the dimensions $\left(\begin{array}{c} N\\ 2 \end{array}\right)$ by 1. Hence, we compute the sample covariance matrix as:

(10) $$\begin{array}{c} C=\frac{1}{M-1}\sum_{i=1}^{M}\left(z_{\text{i}}-\overset{-}{z}\right){\left(z_{\text{i}}-\overset{-}{z}\right)}^{T}, \end{array}$$

where $\overset{-}{z}=1/M\sum_{i=1}^{M}z_{\text{i}}$.

Let the eigenvalues of the $\left(\begin{array}{c} N\\ 2 \end{array}\right)\times \left(\begin{array}{c} N\\ 2 \end{array}\right)$ matrix **C** be denoted by $\lambda_{1},\ldots ,\lambda_{\left(\begin{array}{c} N\\ 2 \end{array}\right)}$ and arranged in decreasing order, $\lambda_{1}\geq \lambda_{2}\geq \cdots \geq \lambda_{\left(\begin{array}{c} N\\ 2 \end{array}\right)}$, so that *λ*_1_ = *λ*_max⁡_. The associated eigenvectors are used to construct an $\left(\begin{array}{c} N\\ 2 \end{array}\right)\times \left(\begin{array}{c} N\\ 2 \end{array}\right)$ matrix V=[v1,…,v(N2)]. We can then write the eigendecomposition equation as **C** **V** = **V**Λ where Λ is a diagonal matrix defined by the eigenvalues of matrix **C**.

In order to ensure minimal redundancy, we need to project the original data set, **z**_1_,…, **z**_M_, onto a few principal components which correspond to the eigenvectors, [**v**_1_,…, **v**_L_], with the largest *L* eigenvalues such that

(11) $$\begin{array}{c} \frac{\sum_{i=1}^{L}\lambda_{i}}{\sum_{i=1}^{\left(\begin{array}{c} N\\ 2 \end{array}\right)}\lambda_{i}}\times 100\geq \xi \end{array}$$

the cumulative energy represented by these principal components account for some certain percentage, *ξ*, of the total energy in the data set. In this paper, we use *ξ* = 90% to obtain a projected set of vectors as

(12) $$\begin{array}{c} p_{\text{i}}={\left[v_{1},\ldots ,v_{\text{L}}\right]}^{T}z_{\text{i}},\;i=1,\ldots ,M. \end{array}$$

The projected vectors are transformed to the original space as

(13) z~i=[v1,…,vL]pi=[v1,…,vL][v1,…,vL]Tzi.

Hence, the new set of vectors, ${\tilde{z}}_{1},\ldots ,{\tilde{z}}_{\text{M}}$, conserves 90% of the total energy within the particular event interval and contains only the most relevant information about the network dynamics. For each event interval, we compute the mean vector:

(14) $$\begin{array}{c} \overset{-}{\tilde{z}}=\frac{1}{M}\sum_{i=1}^{M}{\tilde{z}}_{\text{i}}, \end{array}$$

which will be reshaped such that it constitutes the upper triangular part of the symmetric key graph.

### 3.4. Significance Testing for the Key Graph Estimation

 Since the distribution of the interactions under the null hypothesis which form a key graph for a particular interval cannot be obtained analytically, we resort to generating random networks to derive this distribution. For each key graph extracted for a given time interval, we derived an ensemble of 2000 surrogate time-varying networks by randomly reshuffling the edge weights [15]. The key graph estimation algorithm is applied to each surrogate time-varying graph set in each interval which resulted in 2000 surrogate key graphs. In order to compare the original key graphs with the ones obtained from the surrogate data sets, we selected two different *P*-values, *P* < 0.01 and *P* < 0.001, to determine the significant interactions at 99% and 99.9% significance levels, respectively.

## 4. Data

### 4.1. EEG Data

 To evaluate the performance of the proposed measure in summarizing the event intervals with biological data, we use a set of EEG data containing the error-related negativity (ERN). The ERN is an event-related potential that occurs following performance errors in a speeded reaction time task [47, 48]. The ERN is observed as a sharp negative trend in EEG recordings which typically peaks from 75–80 ms after the error response. Previously reported EEG data from 62 channels were utilized [49]. This study included 90 (34 male) undergraduate students. (Two of the original 92 participants were dropped due to artifacts rendering computation of the PLV values problematic.) Full methodological details of the recording are available in the previous report [49]. The task was a common speeded-response letter (H/S) flanker, where error and correct response-locked trials from each subject were utilized. A random subset of correct trials was selected, to equate the number of errors relative to correct trials for each participant. Before computing the phase-synchrony measures, all EEG epochs were converted to current source density (CSD) using published methods [50, 51]. This was done to accentuate local activity (e.g., to better index small world properties) and to attenuate distal activity (e.g., volume conduction).

There has been longstanding interest in time-frequency representations of the ERN [36, 52, 53]. It has now been established that the time-frequency energy in the ERN occurs in the theta band (4–8 Hz) of the EEG, occurring medial frontally. This activity has been shown to have primary sources in the anterior cingulate cortex (ACC) [54–56]. Observations of similar theta activity across a number of different tasks has been reported, suggesting that midline frontal theta activity may serve related roles across a number of cognitive processes [57]. New attention has been focused on the functional connectivity occurring during the ERN, to better understand the role of medial-frontal theta activity in functional networks subserving cognitive control. Cavanagh and colleagues [58], for example, found evidence that lateral-prefrontal cortex (lPFC) activity was phase synchronous with medial-frontal theta, supporting the idea that medial-prefrontal (mPFC) and lPFC regions are functionally integrated during error processing. By assessing medial-frontal regions active during the ERN in relation to diffusion tensor imaging (DTI), new work has also helped demonstrate how mPFC regions are highly integrated with other prefrontal areas during control processing [59]. Together, advances in this area support the view that medial-frontal sources serve as a central region of activity during error processing, and that phase-synchrony measures of theta activity can index this functional integration. At the same time, work in this area is nascent, and new research into the nature of this functional integration is important. The proposed approach is a graph-based data-driven approach to characterizing functional connectivity, and can offer a new look at network patterns occurring during the ERN. Thus, while the primary aims of the current report are methodological (i.e., developing a method for characterizing time-varying graphs), we hypothesize that the medial-frontal region will play a central functional role during the ERN, and will have significant integration with frontal areas, including lateral frontal. Such findings can offer support that the proposed time-varying graph approach produces effects consistent with current theoretical and empirical work in the field.

## 5. Results

### 5.1. Event Intervals

**Figure 1:**
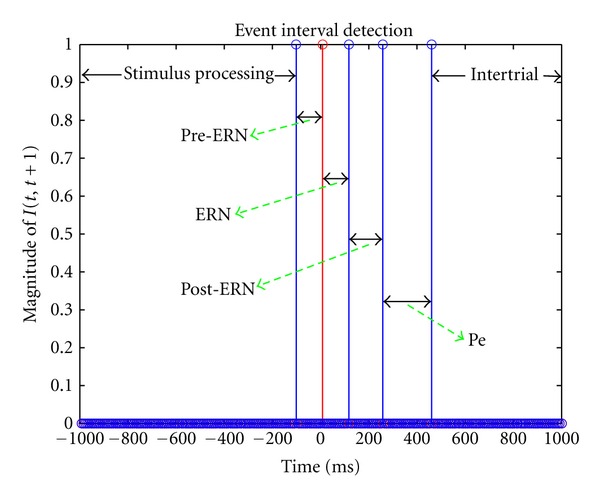
Event interval detection: 6 event intervals are identified which roughly correspond to the stimulus processing (−1000 to −102 ms), pre-ERN (−101 to 0 ms), ERN (1 to 117 ms), post-ERN (118 to 259 ms), Pe (260 to 461 ms), and intertrial (462 to 1000 ms) intervals, respectively. The subjects respond to the stimulus at time 0 ms where the response is represented by the red spike.

 In this paper, we analyzed data from 90 subjects corresponding to the error responses. For each subject, time- and frequency dependent phase synchrony between all possible electrode pairs is computed by RID-Rihaczek-based PLV measure and time-varying graphs, *G*_*t*_^(*q*)^, *t* = 1,…, 256, for the *q*th subject are constructed using (6) where the number of nodes, *N*, is equal to 62, the frequency band of interest is the theta band (4–8 Hz), and the sampling frequency is 128 Hz. Furthermore, a mean time-varying graph sequence, ${\overset{-}{G}}_{t}$, is computed over all subjects as

(15) $$\begin{array}{c} {\overset{-}{G}}_{t}=\frac{1}{90}\sum_{q=1}^{90}G_{t}^{\left(q\right)}, \end{array}$$

and the event interval detection algorithm is applied to this average sequence, ${\overset{-}{G}}_{t}$, where the length of the moving average window, *δ*, is chosen as 2.5% of the sampling period. The value of *δ* is selected such that the window length is able to both detect the abrupt changes in the connectivity patterns and prevent oversmoothing. Different values of moving average window can be chosen depending on the sampling frequency or the application type. We identified 6 different key event intervals based on the proposed change detection algorithm which roughly correspond to the stimulus processing (−1000 to −102 ms), pre-ERN (−101 to 0 ms), ERN (1 to 117 ms), post-ERN (118 to 259 ms), Pe (260 to 461 ms), and intertrial (462 to 1000 ms) intervals, respectively, as shown in Figure 1.

The detected event intervals are consistent with the speeded reaction-time task as the subjects respond to the stimulus at time 0 ms. The first interval indexes complex processing of the imperative stimulus before making a response. The Pre-ERN and Post-ERN intervals, just before and after the ERN, index activity around the incorrect motor response. Importantly, the ERN interval (117 ms time window after the response) and Pe interval (260–461 ms time window) are detected successfully by the event detection algorithm. The Pe (error-positivity) interval corresponds to a P3-like component observed subsequent to the incorrect response [60, 61]. However, measures of P3 energy generally show activity in lower frequency delta bands (e.g., [62–65]), rather than the currently measured theta activity.

### 5.2. Key Graphs

**Figure 2:**
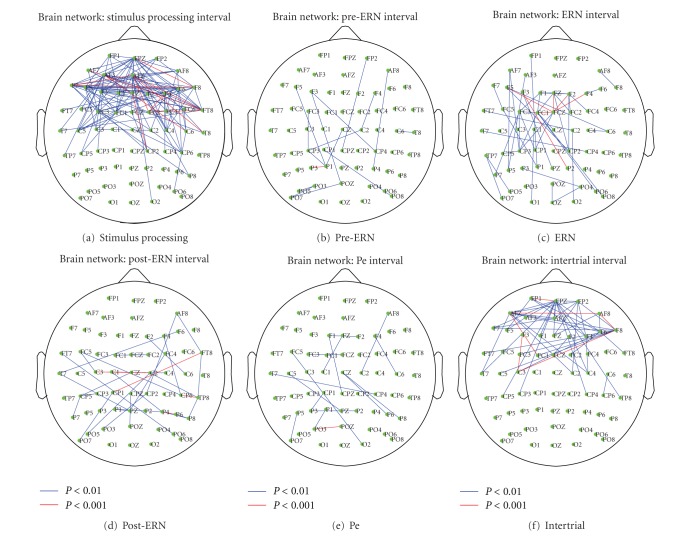
For each event interval, a key graph is obtained using the framework described in Section 3. We compared the extracted key graphs with the ones obtained from the surrogate time-varying graphs and identified the interactions which are significant. Using each key graph, the interactions which are found to be significant at two different levels, *P* < 0.01 and *P* < 0.001, are represented in blue and red colors, respectively.

 For each event interval detected from the mean time-varying graph sequence, ${\overset{-}{G}}_{t}$, *M* vectors, **z**_1_,…, **z**_M_, corresponding to the upper triangular part of the graph, sequences in that interval are formed and the $\left(\begin{array}{c} N\\ 2 \end{array}\right)\times \left(\begin{array}{c} N\\ 2 \end{array}\right)$ covariance matrix is computed as given in (10) where *M* corresponds to the number of graphs that compose the particular event interval and *N* is the number of nodes within the network (*N* = 62). Note that *M* will change for each time interval. For instance, for this particular study *M* = 115 for the stimulus processing, *M* = 13 for the pre-ERN, *M* = 15 for the ERN, *M* = 18 for the post-ERN, *M* = 26 for the Pe and *M* = 69 for the inter-trial intervals. The *L* largest eigenvalues for that event interval are selected such that a 90% energy threshold is satisfied using (11). A corresponding mean vector, $\overset{-}{\tilde{z}}$, which constitutes the upper triangular part of the symmetric key graph for the particular event interval is obtained using (14). Furthermore, we compared the extracted key graphs with the ones obtained from the surrogate time-varying graphs and identified the interactions which are statistically significant as described in Section 3.4. For each event interval, Figure 2 shows the interactions which are significant at two different significance levels where the interactions with *P* < 0.01 and *P* < 0.001 are represented in blue and red colors, respectively. As one can see from Figure 2, ERN interval has much more significant connections compared to the Pre-ERN and Post-ERN intervals as expected because of the complex activity associated with the error commission. In particular, the frontal electrodes (F5, FZ, F2, and F4) have significant connections with the central electrode (FCz) with *P* < 0.001, consistent with previously observed interactions in theta band between medial prefrontal cortex (mPFC) and lateral prefrontal cortex (lPFC) during error-related cognitive control processes [58], whereas the other event intervals do not include such interactions among frontal and central sites. During the Pe, on the other hand, we observe significant connections only among the parietal and occipital-parietal electrodes with *P* < 0.01 and *P* < 0.001. Hypotheses about theta activity during the Pe are underdeveloped in the literature, because P3-related activity generally occurs at lower frequencies (e.g., 0–3 Hz, as described above). Thus, while the observed pattern of effects could be interpreted, it is more reasonable to note that this interval contains the fewest connections between nodes among the identified intervals.

We also focused on the change in connectivity for FCz electrode with the remaining 61 electrodes within the key graphs for Pre-ERN, ERN, and Post-ERN intervals and compared these connectivity values to identify if FCz has stronger connectivity during the ERN interval compared to the Pre-ERN and Post-ERN intervals. We used a Welch's *t*-test at 5% significance level to test the null hypothesis that the connectivity strengths from different key graphs are independent random samples from normal distributions with equal means. For both comparisons, Pre-ERN versus ERN and Post-ERN versus ERN, the null hypothesis is rejected where FCz has a larger mean connectivity for the ERN interval indicating that the central electrode has significantly larger connectivity with the rest of the brain during the ERN interval. Moreover, we compared the connectivity values for Pre-ERN and Post-ERN where there is no significant difference between the connectivity values from these intervals.

## 6. Conclusions

 In this paper, we proposed a new framework to summarize the dynamic evolution of brain networks. The proposed approach is based on finding the event intervals and revealing the informative transient or dynamic interactions within each interval such that the key graph would summarize the particular interval with minimal redundancy. Expectable results from the application to real EEG data containing the ERN supports the effectiveness of the proposed framework in determining the event intervals of dynamic brain networks and summarizing network activity with a few number of representative networks.

Future work will concentrate on exploring different event interval detection and key graph extraction criteria such as entropy-based divergence measures and Bayesian approaches such as the one discussed in [37], which may result in an improved performance in summarizing dynamic networks. Furthermore, the proposed framework will be extended to compare the dynamic nature of functional networks for error and correct responses to get a more complete understanding of cognitive control. In addition, we will employ the proposed framework to analyze data in other frequency bands including delta, which may be more central to activity during the Pe interval. Future work will also consider exploring single-dipole [56, 66] and distributed-dipole [67] source solutions to the inverse problem for extending our proposed dynamic functional connectivity analysis framework to the source domain. Finally, we will explore different group analysis methods to consider the variability across individual subjects and possibly reveal the distinctive network features for each subject rather than averaging the time-varying graphs from all subjects.
